# The volatile flavor and the antioxidant properties of a novel chrysanthemum rice wine during natural aging

**DOI:** 10.1002/fsn3.3247

**Published:** 2023-02-28

**Authors:** Yu Zhang, Huai‐Ning Yin, Ting Zhao, Cheng Zhan, Ai‐Yuan Wang, Yang‐Hui Xu, Mao‐Bin Chen

**Affiliations:** ^1^ Cooperative Innovation Center of Industrial Fermentation (Ministry of Education & Hubei Province), Key Laboratory of Fermentation Engineering (Ministry of Education), Hubei Key Laboratory of Industrial Microbiology, School of Biological Engineering and Food Hubei University of Technology Wuhan China; ^2^ Angel Yeast Co. Ltd Yichang China

**Keywords:** antioxidant, *Chrysanthemum morifolium* cv. Fubaiju, natural aging, rice wine, volatile compounds

## Abstract

*Chrysanthemum morifolium* cv. Fubaiju, a traditional tea in southern China with high nutritional and health functions was used in this study. Optimized production conditions of a novel chrysanthemum rice wine (FRW) were obtained by the Box–Behnken design response surface experiment. FRW with best sensory quality was developed with 0.68% chrysanthemum, 0.79% Jiuqu and 0.81:1 liquid‐to‐solid ratio. Compared with rice wine (RW) control, the total phenolic and flavonoid contents, as well as antioxidant activity of the FRW increased significantly. GC–MS analysis showed that more flavor compounds including alcohols, aldehydes, acids, and esters were detected in FRW. During the aging process, it was found that the antioxidant substances, the antioxidant activity and the flavor substances decreased, with the wine body tending to be homogenized. After 6 months of storage, overall sensory quality of FRW was more harmonious, with special nectar taste, which dramatically improved the flavor characteristics and functionality compared with traditional RW.

## Graphical abstract

Technological process and aging analysis of chrysanthemum rice wine.

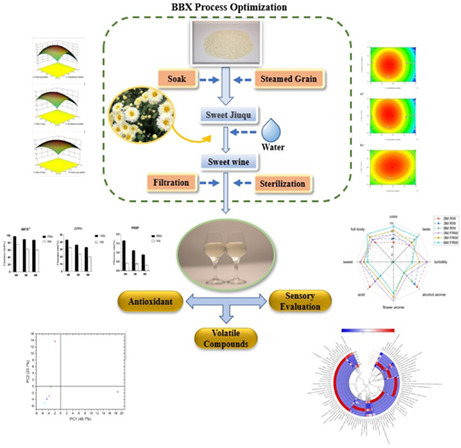

## 1 INTRODUCTION

*Chrysanthemum morifolium* cv. Fubaiju, a perennial plant of the Chrysanthemum family, is a Chinese medicinal herb with a homology of food and medicine. It is called “broad‐spectrum antibiotic” for the higher contents of chlorogenic acid and total flavonoid than other chrysanthemum varieties (Sun et al., 2021). Macheng has a long history of cultivating *C. morifolium* cv. Fubaiju, which dates back to the Song Dynasty. The soil and climate are appropriate for the development of chrysanthemum. After thousands of years of cultivation, the lasting planting range has covered more than 16,000 acres. As green food geographical landmark, Macheng *C. morifolium* cv. Fubaiju has the characteristics of big and thick blossoms, jade white petals, and dull yellow stamens, which contains a variety of chemical components such as flavonoids, phenols, organic acids, volatile oil, and so on, with antioxidant, anti‐inflammatory, and detoxification effects (Cui et al., 2014; Tu et al., 2021).

In recent years, more and more attention has been paid to the relevant research on functional components rich in *C. morifolium* cv. Fubaiju, including different solvent extracts, essential oils, and polysaccharides (Wang, Chen, & Zhou, 2019; Wang, Sun, et al., 2019). In previous studies, hot‐water extract and ethanol extract of *C. morifolium* cv. Fubaiju were obtained (Li, Hao, et al., 2019; Li, Yang, et al., 2019). It was found that hot‐water extract had stronger anti‐inflammatory and free radical scavenging activities than ethanol extract. Hot‐water extract could also increase the abundance of *Bacteroidetes*, *Firmicutes*, *Bifidobacteria*, and *Precotella*, which indicated that drinking chrysanthemum tea might regulate intestinal microorganisms. The water extract of *C. morifolium* cv. Fubaiju was found to have the ability to resist the oxidative damage in ARPE19 cells. Pretreatment with 100 μg/mL water extract could significantly reduce the oxidative damage and apoptosis of ARPE19 cells (Hao et al., 2021). It was reported that the essential oil from *C. morifolium* cv. Fubaiju had certain antifungal effects (Zhan et al., 2021). It might damage not only the cytoplasmic membrane of the fungal cells but also the mitochondria and DNA. The multi‐antifungal mechanisms made it difficult for fungi to develop drug resistance. Recent studies showed that a novel polysaccharide obtained from *C. morifolium* cv. Fubaiju had good emulsifying property and stability (Zhang et al., 2022). However, there is little research on the development of *Chrysanthemum* food products. Chinese rice wine (RW) is a popular traditional refreshing, sweet, and low alcoholic drink, made by short‐term fermentation of glutinous rice and Jiuqu, and is rich in amino acids, organic acids and other nutrients (Zhou et al., 2020). Using functional *C. morifolium* cv. Fubaiju as brewing raw material, the products made will have more nutritional and health care value. It can not only enrich the RW market and improve the added value of products but also provide new ideas for new food production and resource utilization.

**FIGURE 1:**
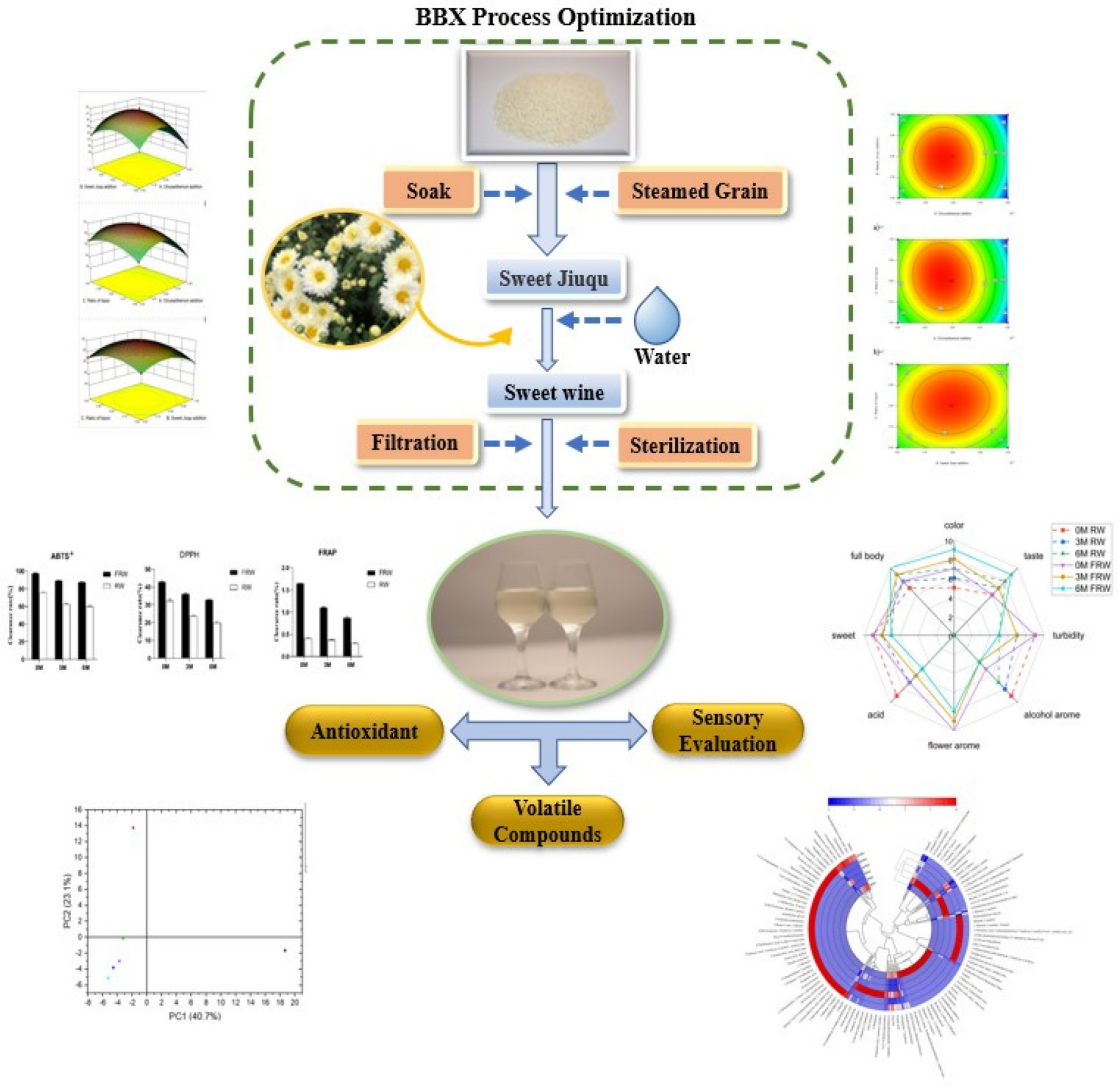
General flow chart of the study.

In this study, a novel *Chrysanthemum* rice wine (FRW) was prepared through response surface optimization experiment. The sensory evaluation, volatile flavor, and antioxidant properties of FRW samples during 6 months of storage were analyzed. The study of the novel FRW can provide clues for further industrial food production, especially for the new alcoholic drinks with flower flavor, nectar taste, and antioxidant function. (Figure 1).

## 2 MATERIALS AND METHODS

### 2.1 Materials

Fine powder of *C. morifolium* cv. Fubaiju (Macheng city, Hubei Province, China), round glutinous rice (Wuchang Caiqiqiao Rice Co., Ltd.), sweet Jiuqu (Angel Yeast Co., Ltd.); 2,2‐Diphenyl−1‐picrylhydrazyl, 2,2′‐Azino‐bis (3‐ethylbenzothiazoline‐6‐sulfonic acid) diammonium salt, 2,4,6‐Tris (2‐pyridyl)‐s‐triazine (Macklin Biochemical Technology Co. Ltd.); sodium chloride, ethanol absolute (analytical grade), potassium persulfate, iron trichloride hexahydrate, sodium acetate trihydrate, glacial acetic acid, aluminum trichloride, potassium acetate, sodium carbonate (Sinopharm Chemical Reagent Co., Ltd.); rutin trihydrate from LGC Labor GmbH; methanol from ThermoFisher Co., Ltd.; folin‐phenol reagent from Solarbio Co., Ltd.

### 2.2 Preparation of chrysanthemum rice wine

#### 2.2.1 Experimental design

Box–Behnken design was used to optimize the production factors of FRW, so as to obtain the overall optimal production process of FRW. Through preliminary exploration of single‐factor orthogonal experiment in the early stage, 17 sets of experiments were conducted with 0.4%–0.8% (dry weight of rice) of chrysanthemum dry weight, 0.6%–1.0% (dry weight of rice) of sweet Jiuqu dry weight, and 0.6:1–1:1 of liquid material ratio as the variables. As shown in Table 1, the three factors selected in this study were designated as X1, X2, and X3, and prescribed into three levels, coded as +1, 0 and −1 (high, medium, and low values) (Jha et al., 2017, 2018). The experimental design is shown in Table 1. The comprehensive score (100 points) was used as the standard for process optimization, including 70% of basic physical and chemical indexes (20% of alcohol content, 15% of total sugar, 15% of total acid, 50% of total flavonoids) and 30% of sensory evaluation.

**TABLE 1:** Variables and levels of Box–Behnken test.

Variables	Levels		
	−1	0	1
Chrysanthemum addition/%	0.40	0.60	0.80
Sweet Jiuqu addition/%	0.60	0.80	1.00
Ratio of liquor	0.6:1	0.8:1	1:1

#### 2.2.2 Preparation and storage of chrysanthemum rice wine

RW and FRW prepared under the optimized process conditions were as follows. One kilogram of glutinous rice was soaked in water for 24 h and steamed for 15 min to make the starch absorbent and gelatinized. Sweet wine koji of 0.79% (dry weight) was added, stirred evenly and placed in a 35 °C incubator for saccharification and fermentation. After 2 days of saccharification, 0.68% of Fubaiju fine powder was added into the fermentation container, and the liquid material ratio was adjusted to 0.81:1. After mixing well, fermentation was carried out under 25 °C for 4 days. During the fermentation process, the content of alcohol (%vol) was measured daily with a digital refractometer. When the alcohol content was 6, it reached the end of fermentation. After filtration and centrifugation, the supernatant was sterilized at 65 °C for 30 min. Under aseptic conditions, the supernatant was evenly divided into several 50‐mL sterile flasks and stored at room temperature for 6 months for further measurement every 3 months.

### 2.3 Determination of total phenolic and total flavonoid

#### 2.3.1 Total phenolic

The determination of total phenolic was according to Li, Hao, et al. (2019) and Li, Yang, et al. (2019) with some modifications. 0.5 mL of FRW was mixed with 2.5 mL of Folin–Ciocalteu reagent, and 7 mL of 20% sodium carbonate was added to start the reaction. The absorbance was measured at 765 nm after a dark reaction at room temperature for 2 h. Using rutin reagent as the standard sample, the standard curve (*y* = 18.86*x* + 0.2378, *R* ^2^ = .9978) was measured according to the above method. The total phenolic content was represented by 1 mg rutin per mL of sample.

#### 2.3.2 Total flavonoids

The total flavonoids were determined according to the method of Zheng et al. (2022) with minor modifications. One microlitre of FRW was mixed with 2 mL of aluminum chloride (0.1 mol/L) and 3 mL of potassium acetate (1 mol/L), and then the solution was fixed to 10 mL with 70% ethanol. After standing for 30 min, the absorbance was measured at 420 nm. Using rutin as standard, the standard curve was measured (*y* = 0.0321*x*‐0.0031, *R* ^2^ = .9977), and the total flavonoid was represented by 1 mg of rutin per mL of sample.

### 2.4 Antioxidant activity assays

#### 2.4.1 DPPH radical scavenging activity

The scavenging activity of DPPH was determined according to the method by Du et al. (2015) with some adjustments. Two‐hundred‐and‐fifty microlitre of the sample was mixed with 5 mL of freshly prepared 0.1 mmol/L DPPH ethanol solution to initiate the reaction, and the absorption at 517 nm was immediately measured as A_S_. After dark reaction at room temperature for 30 min, the solution was centrifuged at 8000 r/min for 5 min to remove impurities precipitate, and the absorption at 517 nm was measured as A_C_. The absorption value of DPPH ethanol solution at 517 nm was A_0_. DPPH radical scavenging activity was calculated by the following formula = [A_*0*_‐(A_*S*_‐A_*C*_)]/A_*0*_ × 100%.

#### 2.4.2 ABTS+ radical scavenging activity

ABTS free radical scavenging activity was determined according to the previously described method (Tian et al., 2018) with some modifications. The ABTS reagent was dissolved in pure water to prepare 7 mmol/L of original ABTS solution, and 2.5 mmol/L of potassium persulfate solution (1:1; V: V) was mixed into the working solution. After 12–16 h of dark storage at room temperature, the working solution was diluted to 0.7 OD at 734 nm for later use. Seven‐hundred‐and‐fifty microlitre of FRW was mixed with 5 mL of diluted working solution, and then placed at room temperature for 6 min. Ac was measured at 734 nm, and A_0_ was obtained in the control group without sample working solution. ABTS scavenging activity was calculated as scavenging rate (%) using the following formula: freeze–thaw rate (%) = (1‐A_*c*_/A_*0*_) × 100%.

#### 2.4.3 Ferric‐reducing antioxidant power

The ferric‐reducing antioxidant power (FRAP) assay was performed according to the procedure (Barreca et al., 2011a, 2011b) with modifications. Acetic acid buffer solution (300 mmol/L, pH = 3.6), TPTZ solution (10 mmol/L in 40 mmol/L HCl), and FeCl_3_·6H_2_O solution (20 mmol/L) were prepared and adjusted to 10:1:1(volume ratio) as working solution for immediate use. One hundred mictolitre of FRW sample was added into 3 mL of working solution, maintained at 37°C for 8 min, and then measured at the absorbance of 593 nm. The standard curve was measured with FeSO_4_ solution, and the reducing capacity of Fe^3+^ was indicated by the concentration of ferrous sulfate solution (mmol/L).

### 2.5 GC–MS analysis of aroma compounds

SPME Fiber assembly polydimethylsiloxane was used in the study. It was the suitable fiber for adsorbing volatile compounds from wines and other alcoholic beverages (Kafkas et al., 2006). The volatile components were identified by Agilent 7890B‐5977B gas chromatography–mass spectrometry (GC–MS). The content of volatile flavor compounds in samples was determined according to the previous method (Liu et al., 2019). Six militre of wine samples and 3 g of NaCl were mixed in a headspace bottle and placed in a thermostatic magnetic stirrer at 55°C for 15 min of balancing (500 r/min). After that, the sample was inserted into the extraction fiber for 30 min of adsorption and analyzed for 70 s. GC conditions: chromatographic column was CP‐WAX57CB (50 m × 0.25 mm × 0.20 μm), temperature programming: with an initial column temperature of 45 °C for 1.5 min, heated to 85 °C at a rate of 6 °C/min, then increased to 225 °C at 4 °C/min, and kept at 225 °C for 15 min. Mass spectrometry conditions: EI source was performed at 150 °C for the quadrupole, 250 °C for the transmission line, and 230 °C for the ion source. Electron energy of 70 eV, full scan mode, scan mass range of 30–550 Aum. Volatile compounds were identified by using chemical standards, retention index, and mass spectral database (Kafkas et al., 2006).

### 2.6 Sensory evaluation

The sensory evaluation team consisted of 20 experts (10 men and 10 women) with professional evaluation experience. The average age of the panelists was 25 years old, and they had received professional training. The tasting room was kept at 25°C, constant temperature and humidity, without foreign odor in the air. According to the evaluation of RW (Yang et al., 2017), the sensory characteristics of the samples were evaluated from eight aspects, including color, turbidity, alcohol aroma, flower aroma, taste, sweetness, acidity, and wine body. In the wine evaluation room, wine samples were divided into professional wine glasses, and each person conducted blind evaluation and scoring on the samples. Three rounds of repeated evaluation were carried out to obtain an average value. The standard score was quantified into five grades (0: none; 1–3: weak; 4–6: medium; 7–9: strong; 10: high intensity), and the results were summarized and plotted on the radar map (Wang et al., 2021).

### 2.7 Statistical analysis

All experiments were replicated at least three times. Data were expressed as mean ± standard deviation (SD). Design‐expert (Version 8.0.6) software was used for experimental design and data analysis; GraphPad Prism 8.2.1 was used for plotting and regression analysis of variance. Tukey test was used for the determination of the significance between the means.

## 3 RESULTS AND DISCUSSION

### 3.1 Sample preparation

Design‐expert 8.0.6 software was used to perform multiple quadratic regression fitting for the experimental results. The variance analysis results of the regression equation are shown in Table 2.

**TABLE 2:** Analysis of variance of response surface regression equation.

Source	Squares	Df	Square	*F*	*p*‐Value	Significance
Model	78.71	9	8.75	58.31	<.0001	**
A‐Chrysanthemum addition	10.12	1	10.12	67.50	<.0001	**
B‐Sweet Jiuqu addition	0.12	1	0.12	0.83	.3917	
C‐Ratio of liquor	0.50	1	0.50	3.33	.1106	
AB	−2.842 E‐014	1	−2.842 E‐014	−1.895 E‐013	1.0000	
AC	0.25	1	0.25	1.67	.2377	
BC	0.25	1	0.25	1.67	.2377	
A^2^	41.78	1	41.78	278.53	<.0001	**
B^2^	11.46	1	11.46	76.42	<.0001	**
C^2^	8.25	1	8.25	55.02	.0001	**
Residual	1.05	7	0.15			
Lack of fit	0.25	3	0.083	0.42	.7510	
Pure error	0.80	4	0.20			
Cor total	79.76	16				

*Note*: (**p < .01, significant difference; *p < .05, significant difference).

It could be seen from the results that the F value of the regression model was 58.31, the significance test was extremely significant (*P* < .0001), and the misfitting item was not significant (*P* = .7510 > .05), indicating that the model was reliable. Through regression fitting of data, the binary regression equation of interaction term and square term of the comprehensive score on the independent variables of chrysanthemum amount, sweet Jiuqu amount, and liquid‐to‐material ratio was obtained:

$$\text{Rice wine comprehensive score}=91.80-1.12\times A-0.12\times B+0.25\times C+0.00\times \mathrm{AB}-0.25\times \mathrm{AC}+0.25\times \mathrm{BC}-3.15\times A^{2}-1.65\times B^{2}-1.40\times C^{2}\;$$

**FIGURE 2:**
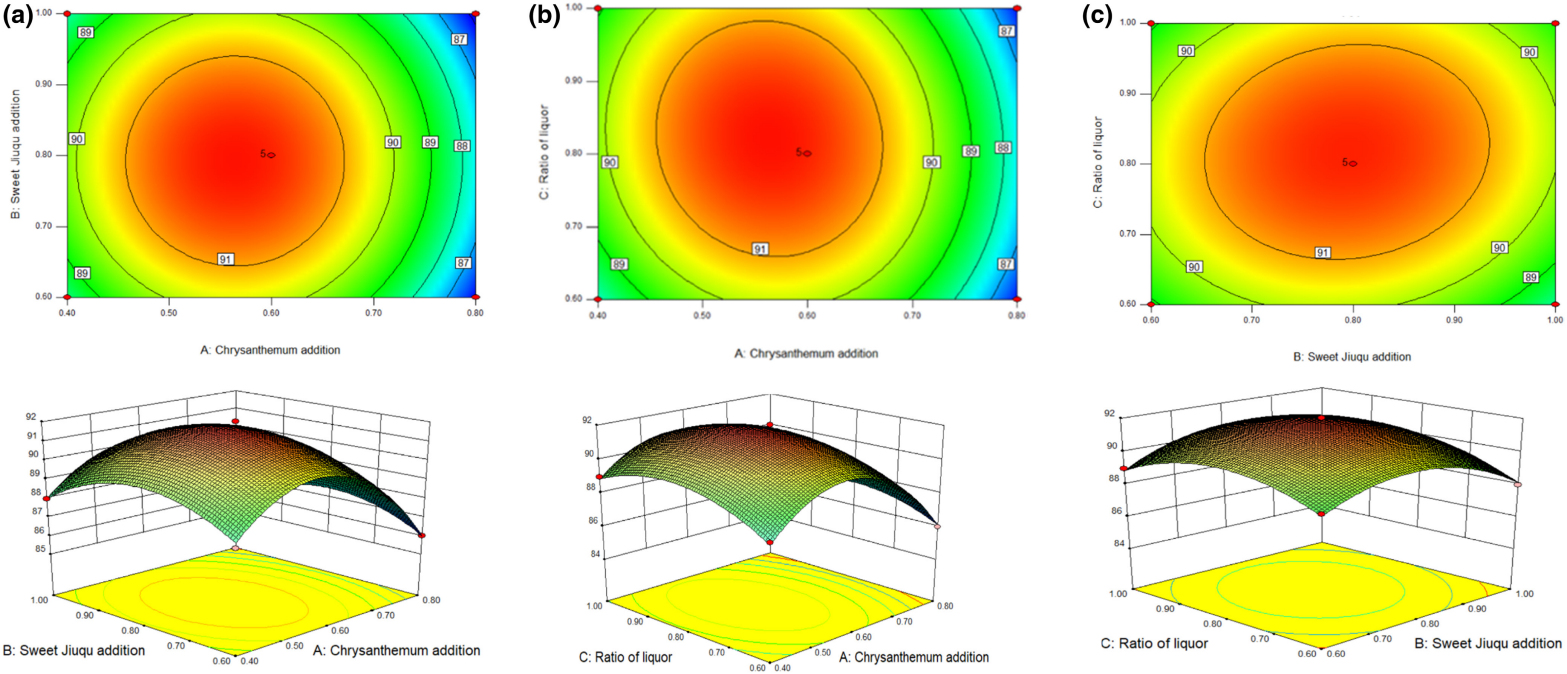
Response surface of the interaction of various factors. *Note*: Response surface and contour plots describing the interactive effects of (a) sweet Jiuqu + chrysanthemum, (b) water + chrysanthemum, and (c) sweet Jiuqu + water.

The determination coefficients of the regression equation *R* ^2^ = .9868 and Adj *R* ^2^ = .9699 indicated that the predicted value and measured value of the model had a good fitting degree and reliability. Design‐expert 8.0.6 software was used to make the quadratic response surface regression model, as shown in Figure 2.

The response surface diagram can directly reflect the interaction between various factors. In this study, there were obvious interactions between the amount of chrysanthemum and sweet Jiuqu, the amount of chrysanthemum and the liquid material ratio, the amount of sweet Jiuqu and the liquid material ratio, which proved the accuracy of response surface curve analysis. According to the above regression model, the optimal production process of FRW was as follows: the amount of chrysanthemum was 0.68% of dry rice weight, the amount of sweet Jiuqu was 0.79% of dry rice weight, and the liquid material ratio was 0.81:1. Under this condition, the FRW comprehensive score predicted by the model was 91.0698. The result of the validation test was close to the predicted value, indicating that the model was well fitted and reliable. Prepared FRW samples were stored for further analysis.

### 3.2 Total phenolics and flavonoids

Total phenolics and flavonoids in food were confirmed to have antioxidant function (Chen, Hui, et al., 2021; Chen, Liu, et al., 2021). Chrysanthemum and glutinous rice in this study were also proved to contain these functional components, which were important indicators for evaluating the functionality of products (Cai et al., 2019). During the aging process (Table 3), the content of total phenolics and flavonoids in RW and FRW decreased rapidly from 0 to 3 months (*p* < .01) and then decreased slowly from 3 to 6 months (*p* < .01). The contents of total phenols and flavonoids in FRW were higher than that in RW. The total flavonoids in FRW were 0.33, 0.273, and 0.263 mg/mL higher than those in RW at 0, 3, and 6 months, respectively. Previous reports showed that antioxidant activity of flavonoids in plants could prevent damage to the human body by scavenging free radicals (Shen et al., 2022). The results showed that the total flavonoids, phenols, and other substances in chrysanthemum and glutinous rice could be the main sources of free radical scavenging activity and antioxidant activity of FRW samples.

**TABLE 3:** Analysis of total phenol, total flavonoid, and antioxidant activities of rice wine (RW) and chrysanthemum rice wine (FRW) at different aging stages

Index	Sample	Time		
		0 M	3 M	6 M
Total phenolic (mg/L)	RW	0.106 ± 0.001^Aa^	0.088 ± 0.001^Ab^	0.079 ± 0.001^Ac^
	FRW	0.111 ± 0.001^Ba^	0.084 ± 0.001^Bb^	0.083 ± 0.002^Bc^
Total flavonoid (mg/L)	RW	0.224 ± 0.003^Aa^	0.213 ± 0.007^Ab^	0.196 ± 0.009^Ac^
	FRW	0.554 ± 0.017^Ba^	0.486 ± 0.015^Bb^	0.459 ± 0.01^Bc^
DPPH (%)	RW	46.49% ± 0.92%^Aa^	36.14% ± 3.10%^Ab^	29.5% ± 1.08%^Ac^
	FRW	33.44% ± 0.37%^Ba^	23.60% ± 0.59%^Bb^	18.33% ± 2.01%^Bc^
ABTS (%)	RW	97.49% ± 0.47%^Aa^	89.30% ± 0.57%^Ab^	87.34% ± 0.73%^Ac^
	FRW	75.47% ± 0.62%^Ba^	62.26% ± 0.96%^Bb^	60.18% ± 0.47%^Bc^
FRAP (mmol/L)	RW	1.647% ± 0.006%^Aa^	1.097% ± 0.023%^Ab^	0.870% ± 0.033%^Ac^
	FRW	0.414% ± 0.014%^Ba^	0.376% ± 0.01%^Bb^	0.302% ± 0.003%^Bc^

*Note*: Data are expressed as means ± SD (*n* = 3). Different superscripts (A and B) represent significant differences in the same column (*p* < .01). Different superscripts (a–c) represent significant differences in the same row (*p* < .01). (M‐months).

### 3.3 Antioxidant activity assays

**FIGURE 3:**
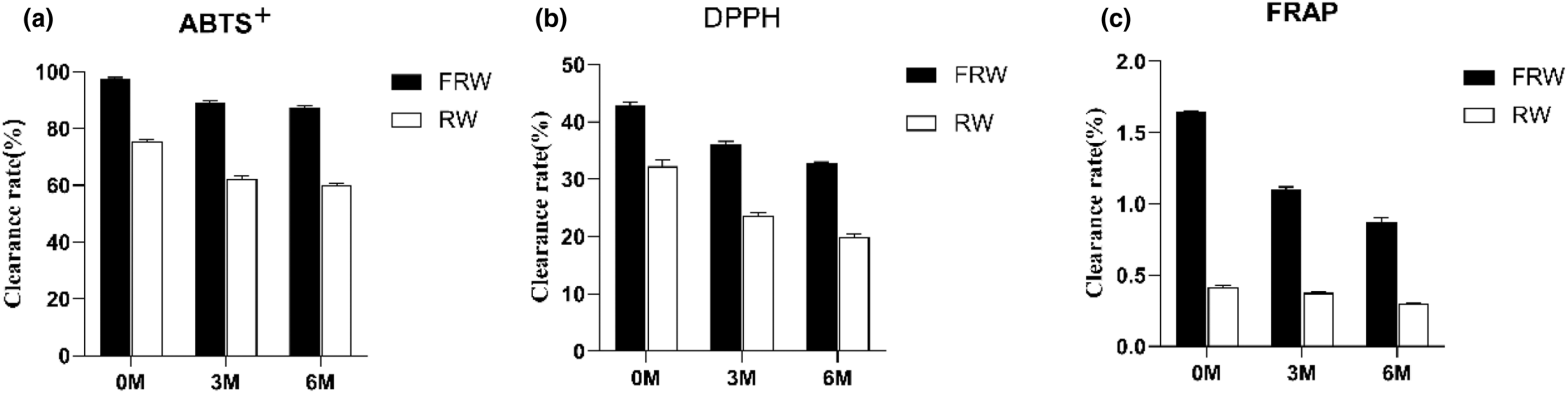
Change trend of ABTS^+^ (a), DPPH (b), and FRAP (c) antioxidant activities during aging process. (M‐months).

According to the free radical scavenging capacity of DPPH, ABTS^+^, and FRAP, the antioxidant capacity of RW and FRW at different aging stages (0, 3, 6 M) was evaluated (Table 3). As shown in Figure 3b, with the increase of aging time, the DPPH scavenging activity of the samples decreased gradually (*p* < .01), but the scavenging rate of FRW was 13.05%–11.17% higher than that of RW. Previous studies found that the increase of total phenol in Cuban fruit wines was positively correlated with the increase of free radical scavenging activity (Núñez et al., 2021), which was consistent with the results of this study, indicating that the addition of chrysanthemum would promote the increase of free radical scavenging activity in FRW.

It can be seen from Figure 3a that ABTS^+^ free radical scavenging activity also decreased with aging time (*p* < .01), but the free radical scavenging activity of FRW at 0 month was as high as 97.49% ± 0.47% and that of RW was 75.47% ± 0.62%. After aging for 3 months, it decreased to 89.30% ± 0.57% (FRW) and 62.26% ± 0.96% (RW). After aging for 6 months, there were 87.34% ± 0.73% (FRW) and 60.18% ± 0.47% (RW). The free radical scavenging activity of ABTS^+^ was much higher than that of DPPH, which might be due to different quenching mechanisms. DPPH is a neutral nitrogen‐containing radical, while ABTS is a positive nitrogen‐containing radical, which indicates that the components in the sample are more effective for positive radicals (Barreca et al., 2011a, 2011b; Tian et al., 2018). In addition, the activity in DPPH assay was from flavones rather than flavanones (Hua et al., 2021). Therefore, it could be inferred that the content of total phenol and flavanone in the sample is higher than that of flavones, and the decreased rate of total flavonoids and phenols during the storage period is positively correlated with the decrease of free radical scavenging ability of the sample.

As shown in Figure 3c, the total antioxidant activity of FRW was significantly higher than that of RW (*p* < .01), which might be the reason for adding chrysanthemum. The addition of Fu Brick tea improved the reducibility of traditional glutinous RW, which might provide a reference for the study (Xu et al., 2019). The iron ions’ reduction capacity of RW and FRW samples showed a downward trend in the aging process (*p* < .01). At 0 month, the total antioxidant capacity of FRW was 3.97 times that of RW. After aging for 3 and 6 months, the total antioxidant activity of FRW decreased faster than that of RW. However, the total value of FRW was much higher than that of RW, reaching 2.92 times (3 M) and 2.88 times (6 M), respectively, indicating that adding 0.6% Chrysanthemum powder provided FRW with the main antioxidant activity, but it might be easier to lose its antioxidant activity than RW during storage.

### 3.4 GC–MS identification of aroma compounds

**FIGURE 4:**
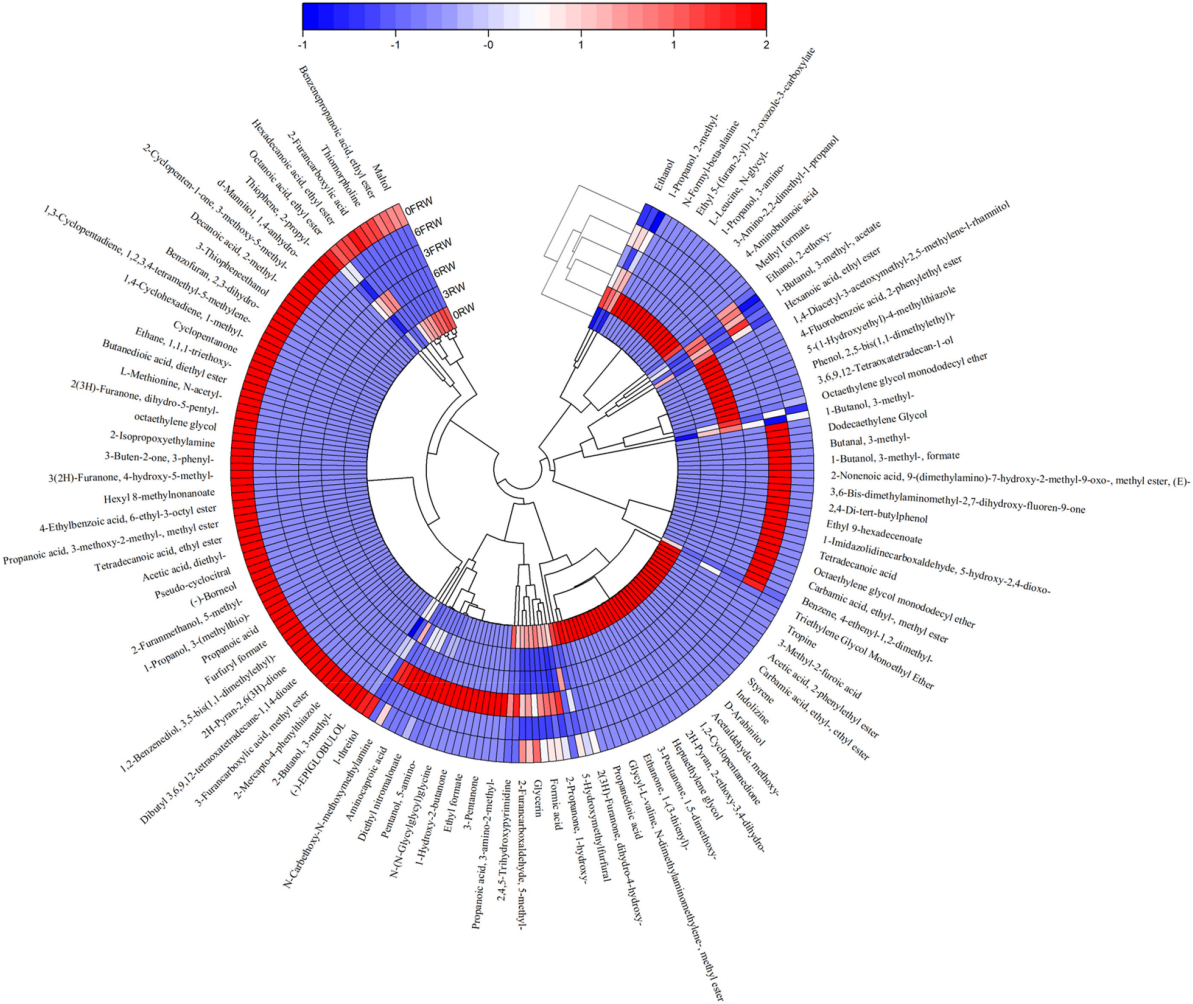
Thermogram of volatile components of different samples during aging.

Headspace solid‐phase microextraction gas chromatography–mass spectrometry (HS‐SPME‐GC–MS) was used in this study for the identification of aroma compounds in samples. A total of 104 flavor components were detected in RW, with 148 compounds identified in FRW (Figure 4). In previous studies, 71 volatile organic compounds were found in RW, with 49 potential aging markers (Wang, Chen, & Zhou, 2019; Wang, Sun, et al., 2019). In this study, the different flavor components between RW and FRW might be caused by the addition of chrysanthemum. Because volatile components from chrysanthemum were detected in FRW, such as borneol, isoborneol, N‐decanoic acid, and trans‐chrysanthemic acid (Xiao et al., 2016). In the natural fermentation process of RW, fragrant RW was produced through microbial action and a series of chemical reactions. Chrysanthemum could not only provide the special flavor components of itself into the RW during the fermentation process but also create new flavors.

Octanoic acid, phenoxyl alcohol, 2‐methyl‐1‐propanol, benzaldehyde, 3‐methyl‐1‐butanol, ethanol, lauric acid, and other flavor substances were detected in both RW and FRW during aging time. Among these, phenoxyl alcohol was a unique flavor substance in RW, which could bring rose aroma; 3‐methyl‐1‐butanol with the aroma and spicy taste of apple brandy; benzaldehyde with the special smell of bitter almonds (Chen, Hui, et al., 2021; Chen, Liu, et al., 2021; Yang et al., 2022). As for the flavor markers of RW, they existed in the whole aging stage in a typical style, without changing after storage.

There were 17 flavor components in both 0 month samples of RW and FRW, which could be labeled as the same flavor substances in this period. In addition, there were 124 different flavor components, such as benzene, 4‐methyl‐1‐(1, 5‐dimethyl‐4‐hexenyl), 3(2H)‐furanone, 5‐methyl‐4‐hydroxy, and so on (Zhan et al., 2021), which were the unique flavor components of chrysanthemum. It could be concluded that the special flavor components brought by chrysanthemum might be formed by the interaction between chrysanthemum and microbial fermentation. In 3 months RW and FRW samples, the 5‐aminovaleric acid, 3‐amino‐2‐methylpropanoic acid, aminocaproic acid, and ethyl glycolate were the unique flavor substances, with a greater decrease than 0 month.

There were five typical flavor components in 6 month RW and FRW samples, including hexanoic acid, ethyl caproate, isoamyl acetate, ethyl acetate, and ethyl lactate. During the aging process, the synthesis and decomposition of esters were carried out at the same time, with many macromolecular esters newly formed after aging, such as ethyl acetate, ethyl lactate, and so on. They were the markers of aging RW, and the number of acids and alcohols decreased during aging. In previous studies, similar results were also found that esterification reaction of acids and alcohols resulted in the increase of esters during storage, and the newly synthesized aging markers ethyl 2‐methylpropanoate, ethyl 2‐methylbutanoate, and ethyl nicotinoate were not detected in fresh liquor (Wang, Chen, & Zhou, 2019; Wang, Sun, et al., 2019).

It can be seen from Figure 4 that during the aging process of RW and FRW, the types and quantities of alcohols, acids, and aldehydes decreased rapidly from 0 to 3 months, and gradually decreased from 3 to 6 months, while the types of esters decreased first and then increased (Ni et al., 2021). Ketones, phenols, ethers, alkenes, and other flavor components decreased significantly from 0 to 3 months and balanced from 3 to 6 months. It could be inferred that the RW experienced a series of complex chemical changes in the aging process. During aging, there was a trend of increasing esters and decreasing alcohols and acids, which was consistent with the change of flavor characteristics of the aged yellow RW in the previous report (Yu et al., 2019). Similar results were also found (Yu et al., 2022) that in grape wine, the main aroma components, that is, esters, increased after aging. In this study, in the different aging stages of RW and FRW, there were not only common unchanged flavor substances of RW but also unique flavor components at different aging stages. In 0 month of the fresh RW, the acids, ester, and other flavor components were from the fermentation of rice and chrysanthemum (Chen et al., 2013). After aging, some flavor substances decreased or disappeared, and some new flavor components were generated. The generation and change of these substances were the signs of the aging of RW and the key to the change of wine flavor.

**FIGURE 5:**
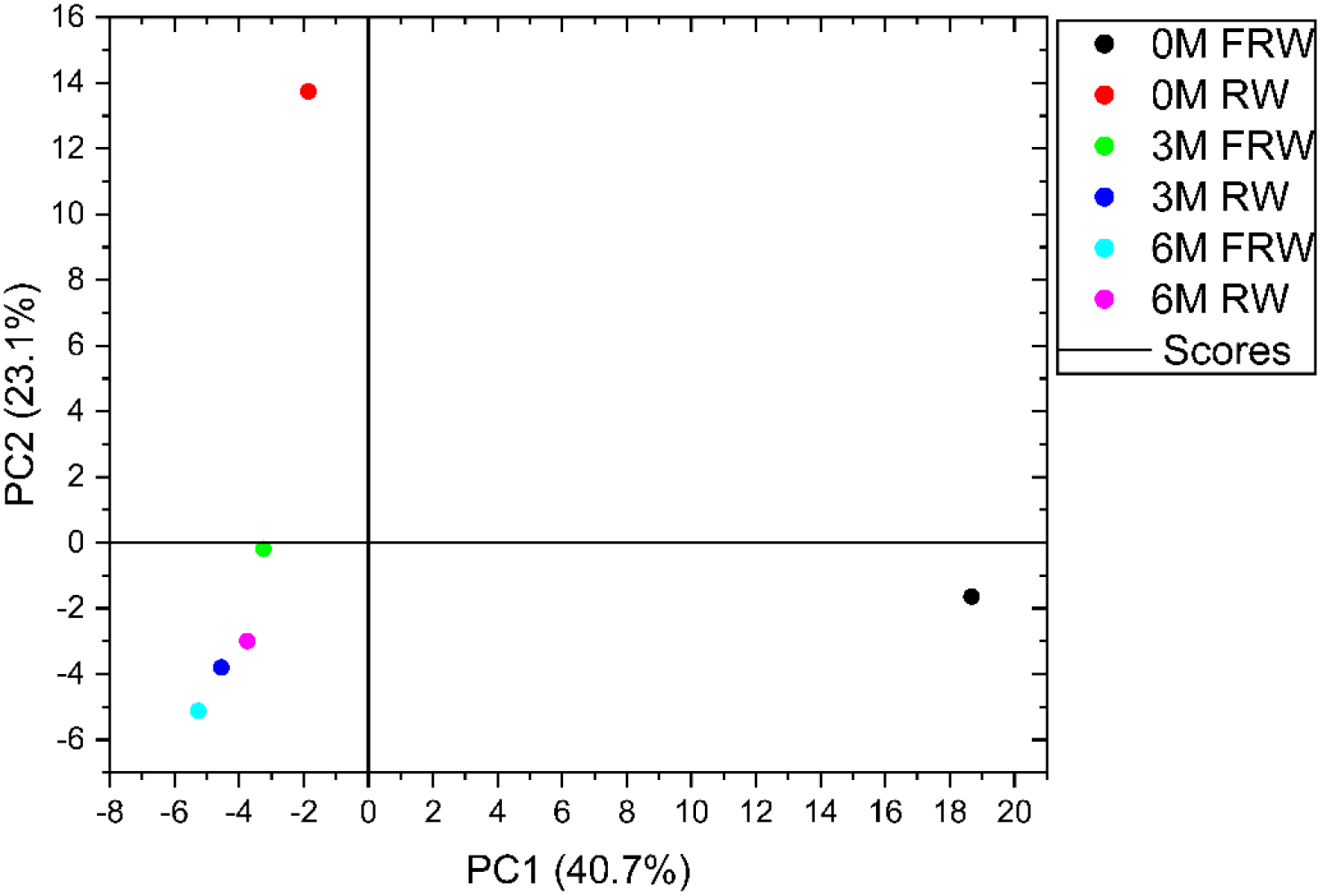
Principal component analysis of rice wine and chrysanthemum rice wine during aging process. (M‐months).

Compared with RW and FRW, the flavor substances produced at the same aging stage were relatively similar, and FRW generated more new flavor substances at the same stage, which was brought by the specific chrysanthemum flavor. In addition, an interesting phenomenon was found in the research based on the Cluster analysis of flavor components, as shown in Figure 5. It was found that the difference between FRW and other samples of 0 month was the biggest and was classified as a separate category, while the difference between the aged FRW wines gradually narrowed. The homogenization of flavor substances in aged wine still needed further research.

### 3.5 Sensory evaluation

**FIGURE 6:**
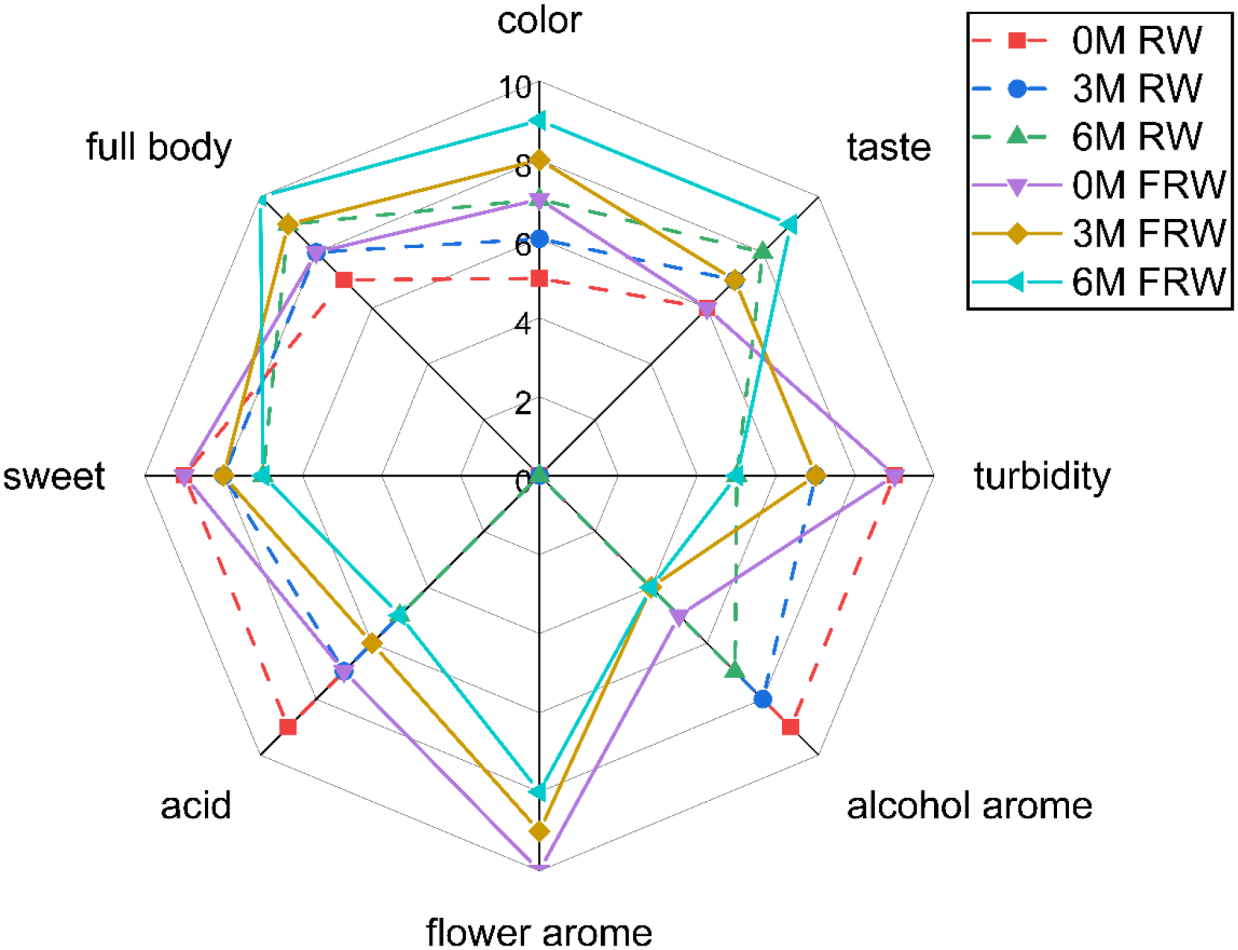
Radar chart of sensory evaluation of rice wine and chrysanthemum rice wine during aging process. (M‐months).

Sensory evaluation was carried out to analyze the sensory characteristics of RW and FRW at different aging stages. The evaluation team evaluated the samples in terms of color, turbidity, alcohol aroma, flower fragrance, taste, acidity, sweetness, and wine body. As shown in Figure 6, RW was yellowish transparent, and the wine body was clear and bright. By adding chrysanthemum, the natural yellow pigment was dissolved in the liquor to make the wine yellow. In the aging process, the yellow color gradually faded, the turbidity increased, and the wine body became turbid and lusterless, which might be related to the oxidation or degradation of some pigment substances and the polymerization precipitation of sample substances during storage.

Fresh RW (0 month RW) was elegant, with the mellow smell of RW, while the flower flavor in fresh FRW (0 month FRW) covered most of the alcohol aroma. In the late aging process, the aroma of FRW gradually weakened, which was positively related to the quantity of the former flavor components. In terms of taste, the 0 month of FRW was sour and sweet, with inharmonious taste. After aging, the sour and sweet taste was weakened, and a more harmonious feeling was obtained. FRW has a sweet nectar taste after 6 months of aging, which indicated that the taste of FRW could be better integrated during aging, making the wine body more balanced. Therefore, adding chrysanthemum of appropriate concentration to glutinous RW has great potential for producing new alcoholic beverages with new sensory characteristics (Wang et al., 2021). Although its aroma and color were insufficient, the 6 months FRW was considered to be the best, with coordination, unique nectar flavor and better overall style. It could be predicted that the chemical components in FRW were constantly in balance during aging, and the esters, volatile phenolic, terpenes, etc. were mixed and correlated with other compounds to enhance the special taste (Culleré et al., 2009; Furtado et al., 2021).

## 4 CONCLUSION

To improve the functionality of traditional Chinese RW, the novel FRW and its volatile aroma during aging were studied. The determination of total flavonoids and phenols, total antioxidant activity (FRAP), free radical scavenging rate (ABTS^+^, DPPH), flavor components analysis, and sensory evaluation in FRW samples were analyzed. The differences in taste, flavor, and function between FRW and RW optimized by Box–Behnken design process were compared. The results showed that the FRW fermented by glutinous rice and chrysanthemum could display better RW flavor, sensory, as well as functional ingredients (Liu et al., 2019). During the aging process, the types of flavor substances and the content of functional components of FRW changed significantly, dropping rapidly from 0 to 3 months and slowly from 3 to 6 months. However, during aging process, the taste was more harmonious. The fusion of chrysanthemum and rice fully produced a unique nectar taste in the final FRW. In a word, the study can provide some reference for developing new functional RW products in the future.

## CONFLICT OF INTEREST STATEMENT

The authors declare no conflict of interest.

## Data Availability

The data that support the findings of this study are available from the corresponding author upon reasonable request.
